# The science module in the MHH’s model curriculum – development, implementation, results

**DOI:** 10.3205/zma001783

**Published:** 2025-11-17

**Authors:** Volker Paulmann, Marie Mikuteit, Ingo Just, Naomi Karmann, Sandra Steffens

**Affiliations:** 1 Hannover Medical School, Dean's Office – Teaching and Learning Research, Hannover, Germany; 2 Hannover Medical School, Institute of Toxicology, Hannover, Germany; 3 Cologne, Germany

**Keywords:** medical studies, scientific skills, curriculum development, NKLM

## Abstract

**Objective:**

At the Hannover Medical School (MHH), a compulsory science module was introduced into the medical curriculum for the 2020/2021 academic year. This article presents the didactic premises – with a focus on teaching scientific skills and critical thinking – and describes their implementation in the form of a longitudinal teaching concept with a final research paper. Results on the students' learning gains are reported against this background.

**Methods:**

The National Competence-Based Learning Objectives Catalogue for Medicine (NKLM) and the results of curriculum mapping were used to identify learning objectives and teaching formats. Learning gains in the newly designed module were assessed using student competence assessments. The research paper concept and initial results are evaluated on the basis of around 200 papers submitted. Furthermore, student feedback (N=81) was collected and analysed using an online questionnaire that was administered after the submission of the research paper.

**Results:**

The science module was implemented as a longitudinal teaching concept. The learning objectives are taught through electronic assessment portfolio tasks (ePF tasks), seminars and a final research paper. Formative feedback is provided to assess the ePF tasks. Around 90% of a cohort successfully completed the tasks and achieved significant learning gains. To date, around 200 students have successfully completed a 10-15 page research paper. They consider the acquisition of scientific writing skills and the ability to conduct structured literature research to be the greatest learning gains.

**Conclusion:**

The introduction of additional components of scientific training offers numerous opportunities and challenges. Teaching the relevant skills is resource-intensive, as teaching usually requires smaller supervision ratios. To strengthen the feedback culture in medical education, little-used teaching methods such as assessment portfolios and research papers can be employed.

## Zusammenfassung

**Zielsetzung:**

An der Medizinischen Hochschule Hannover (MHH) wurde zum Studienjahr 2020/2021 im Medizinstudium ein verpflichtendes Wissenschaftsmodul eingeführt. Der Beitrag stellt die didaktischen Prämissen vor – im Mittelpunkt stehen die Vermittlung von wissenschaftlichen Kompetenzen und kritischem Denken – und beschreibt deren Umsetzung in Form eines longitudinalen Lehrkonzeptes mit einer abschließenden Forschungsarbeit. Ergebnisse zum Lernzuwachs der Studierenden werden vor diesem Hintergrund berichtet.

**Methoden:**

Für die Identifizierung von Lernzielen und Unterrichtsformaten wurden der NKLM sowie Ergebnisse eines Curriculum-Mappings genutzt. Der Lernzuwachs im neu konzipierten Modul wurde anhand von studentischen Kompetenzeinschätzungen erhoben. Das Konzept der Forschungsarbeit und erste Ergebnisse werden auf der Basis von rund 200 vorliegenden Arbeiten evaluiert. Zudem wurden studentische Rückmeldungen (N=81) ausgewertet, die nach Abgabe der Forschungsarbeit mittels eines Online-Fragebogens erhoben wurden.

**Ergebnisse:**

Das Wissenschaftsmodul wurde als longitudinales Lehrkonzept implementiert. Im Rahmen von elektronischen Assessment-Portfolio-Aufgaben (ePF-Aufgaben), Seminaren und einer abschließenden Forschungsarbeit werden die Lernziele vermittelt. Für die Bewertung der ePF-Aufgaben werden formative Feedbacks erstellt. Rund 90% eines Jahrgangs haben die Aufgaben erfolgreich bearbeitet und dabei einen signifikanten Lernerfolg erzielt. Die Erstellung einer Forschungsarbeit im Umfang von 10-15 Seiten haben bislang rund 200 Studierende erfolgreich abgeschlossen. Diese betrachten den Erwerb von wissenschaftlicher Schreibkompetenz und die Fähigkeit zur strukturierten Literaturrecherche als größten Lernzugewinn.

**Schlussfolgerung:**

Die Einführung zusätzlicher wissenschaftlicher Ausbildungselemente bietet zahlreiche Chancen und Herausforderungen. Die Vermittlung der entsprechenden Kompetenzen ist ressourcenintensiv, da die Lehre in der Regel kleinere Betreuungsschlüssel erfordert. Um die Feedbackkultur in der medizinischen Ausbildung zu stärken, können wenig genutzte Lehrformen wie das Assessment-Portfolio, aber auch die Forschungsarbeit, eingesetzt werden.

## Introduction

The premises developed in the “Master plan for medical studies 2020” for the reorientation of the German Medical Licensing Regulations are currently undergoing political negotiations with an uncertain outcome, following the submission of a draft bill [1]. Regardless of the slow legislative implementation, many medical faculties are already implementing the planned study reform in their teaching. With a view to teaching scientific skills in particular, a wide range of teaching approaches has developed in medical education in recent years [2]. These range from selective, propaedeutic offerings in the form of seminars or lectures [3], to extracurricular teaching concepts [4], elective subjects and integrated teaching concepts within the framework of compulsory modules [5], to longitudinal, didactically complex curricula with student research projects as final theses [6], [7], [8], [9]. The National Competence-Based Learning Objectives Catalogue for Medicine (NKLM) already provides comprehensive starting points for the content design of corresponding teaching formats. Despite the existing momentum, development is comparatively slow in international comparison [10]. For example, the principle of a compulsory written thesis in medical studies was established in Austria in 2006 and in Switzerland in 2009 as part of the introduction of a Bachelor's/Master's structure [11]. In Germany, it seems that discussions about medical doctorates have long obscured the view of necessary new curricular approaches [12], [13]. Studies have shown that the doctoral phase is associated with an increase in competence [14], [15]. However, with the focus on independent research, which is the goal of a medical dissertation, the question of what fundamental scientific expertise all doctors should acquire during their studies [16] – and which didactic approaches are appropriate here – seems to have been pushed into the background. Diverse upheavals in the healthcare system are to be expected, particularly in view of the exponential growth of medical knowledge, but also due to the novel information and data processing modes of digitalisation and the development of artificial intelligence [17], [18]. These transformation processes increasingly require doctors to be able to identify reliable sources of information, recognise and assess medical – but also social – developments, and be willing to adapt their own actions. This cycle of reflection is often defined as “lifelong learning” and is closely linked to the basic profile of the critical-analytical scientific “personality” in the CanMEDS roles [19]. From a medical education perspective, it is clear that lectures and multiple-choice exams can only contribute to a limited extent to strengthening this role, as they usually focus on factual knowledge or on procedural and justificatory knowledge [20]. Instead, practice-oriented or research-based teaching and learning contexts are recommended [21]. In the context of a new licensing regulations, a written research paper to be completed over a period of 12 weeks is proposed as a relevant assessment. 

The Hannibal model curriculum was introduced at the Hannover Medical School (MHH) in the 2005/2006 academic year. The curriculum is based on the training ideal of the existing Medical Licensing Regulations, whose goal is to produce “doctors who are scientifically and practically trained in medicine” [https://www.gesetze-im-internet.de/_appro_2002/BJNR240500002.html]. A review after fifteen years showed that the practice-oriented elements in particular had been well developed [22]. In order to strengthen academic qualifications in a similar way, preparatory lectures with an explicit scientific focus were developed in 2017. These were offered on an optional basis, as was the opportunity to write a supervised written thesis. However, student response to these offerings remained lukewarm, prompting the development of new curricular approaches. 

This article presents the newly designed science module at the MHH, which was introduced at the beginning of the 2020/2021 academic year as a compulsory module for 320 students per academic year. It extends from the first to the fifth year of study and concludes with a compulsory research paper. The premises guiding the development and the concrete implementation of the science module are presented below. Against this background, the following research questions will then be answered:

- Do the teaching and learning formats introduced (ePortfolio tasks, seminars, research paper) show an increase in competence among participants?
- Looking at the completed research papers of the first cohort, what conclusions can be drawn from the results and experiences regarding the added value and integration into medical studies?

## Project description

### Development of the science module at the MHH

The expansion of the course offerings for the development of scientific skills was developed by the curriculum development department in the Dean’s Office with the involvement of students and essentially comprised the following steps:

1. Initially, the mapping process carried out at the MHH in line with the National Competence-Based Learning Objectives Catalogue for Medicine (NKLM 1.0) was used to identify the science-related learning objectives that had not yet been taught.
2. Subsequently, in line with *constructive alignment,* teaching formats were assigned to the learning objectives and the examination modalities for the module and its elements were defined.

The module structure was designed with two guiding principles in mind: on the one hand, the desire for a flexible basic structure that would give students individual freedom within a highly structured curriculum. On the other hand, the human and spatial resources of the teaching staff were to be used efficiently so that digital learning and teaching formats could be implemented to a greater extent. The review of the learning objectives for scientific skills (version 1.0: chapter 14a) based on the NKLM and the mapping of the MHH curriculum made it clear that the learning objectives relating to research and critical reflection in particular had not been adequately represented in the curriculum to date. In order to specifically integrate these in medical studies, the following teaching elements were implemented in the science module.

### ePortfolio tasks

**Figure 1:**
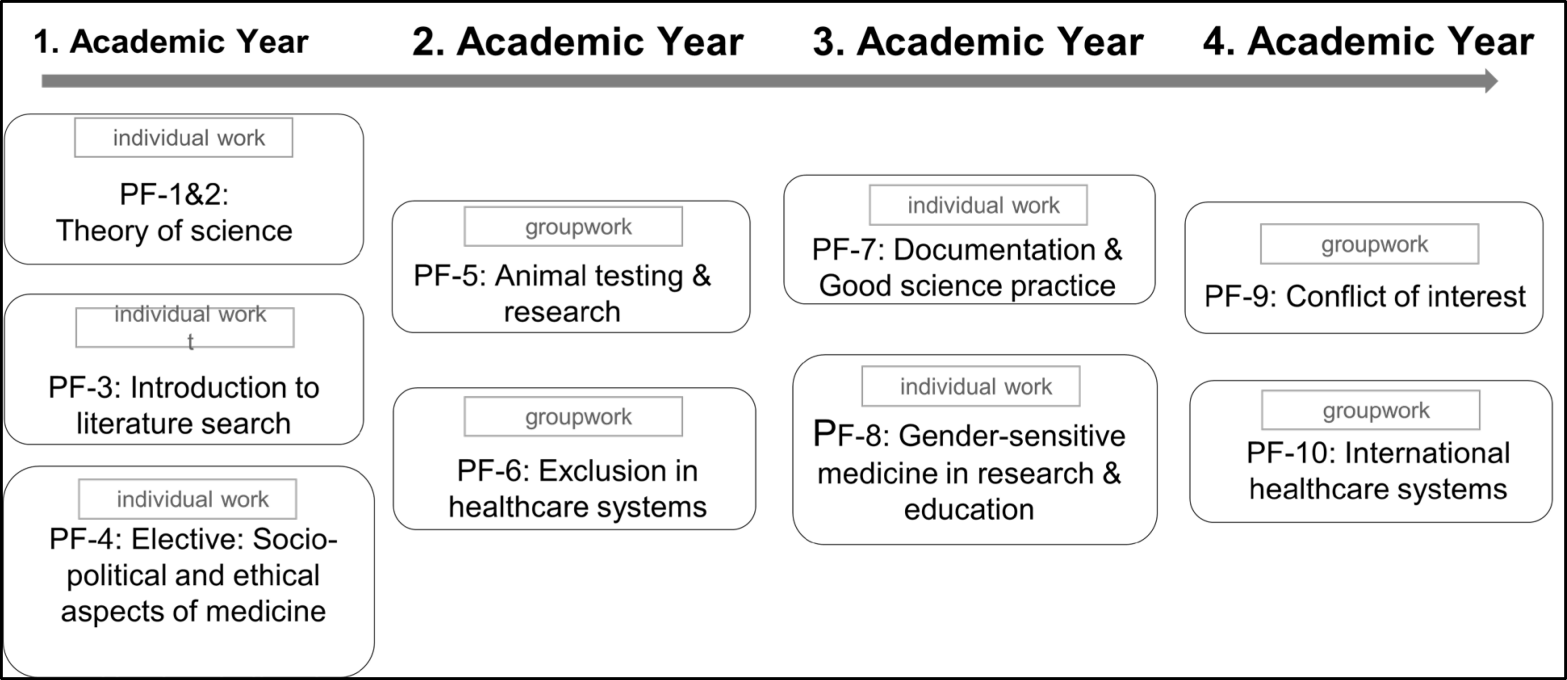
Overview of topics and working modes (individual work vs. group work) for ePortfolio assignments in academic years 1-4. Each assignment has a completion time of between 4 and 8 weeks until the submission deadline.

In order to anchor critical thinking, source analysis and scientific writing more firmly, the little-used assessment portfolio was selected from the MHH’s teaching and exam regulations for the assessment of learning performance. This is defined as “[...] course-accompanying, written performance assessment. It serves primarily to review the development of skills and abilities as well as attitudes and mindsets. [...]” [23]. The basic idea behind this is to set students 2-3 assessment portfolio tasks each year in a longitudinally anchored module format (in academic years 1-5), which they are to complete independently in writing over a period of 4-8 weeks. The tasks, which include both individual and group assignments, are completed digitally via the ILIAS learning platform so that, similar to electronic examinations [24], they are referred to as “ePortfolio tasks (ePF tasks)” and, in their entirety, as “eAssessment portfolio (ePF-A)”. The topics covered include aspects of science theory, literature search and good scientific practice (GWP), as well as questions relating to development processes in the healthcare system (see figure 1 (Fig. 1)). All tasks are tested by students in advance to remove any barriers to understanding and to obtain estimates of the time required to complete them.

**Figure 2:**
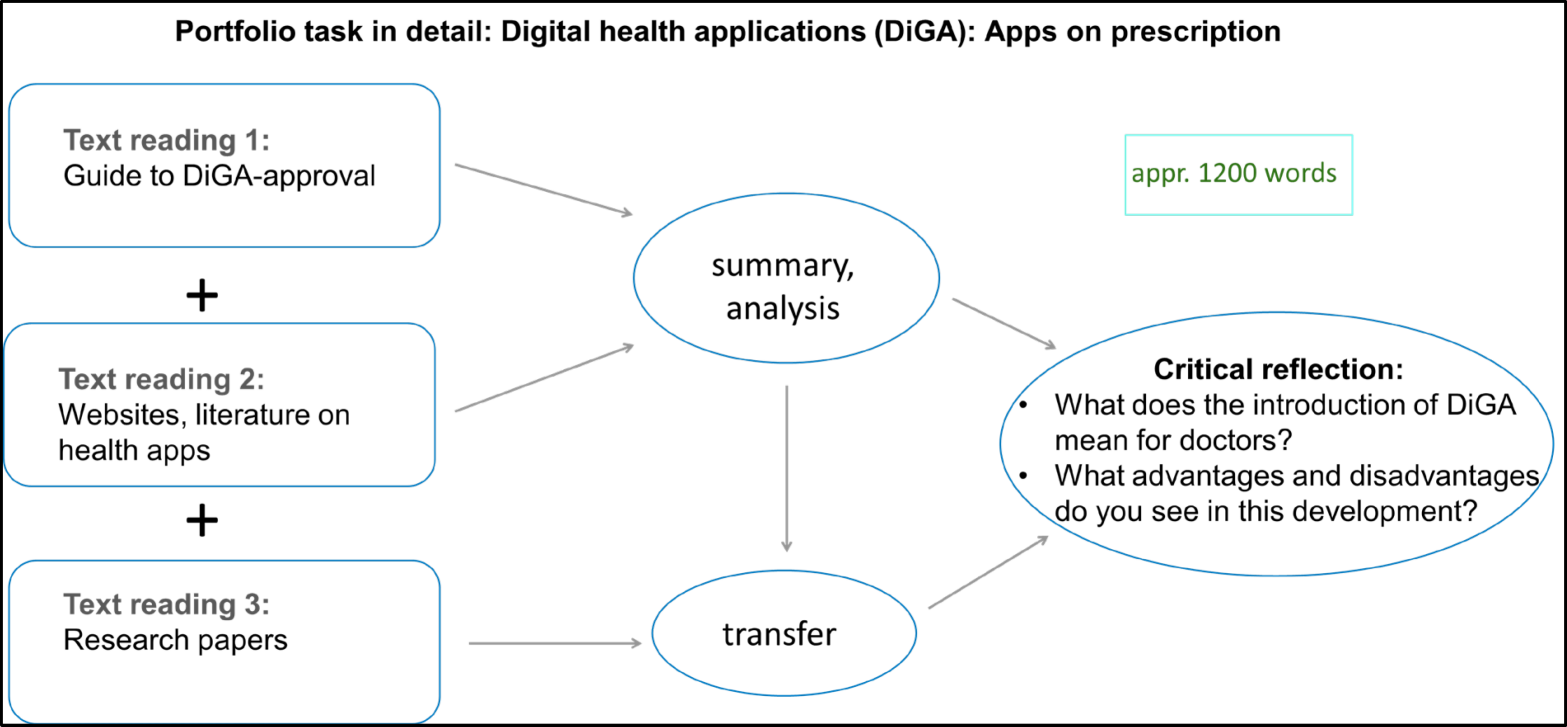
Example of the didactic structure of an ePortfolio assignment (1^st^ year of study) Using the example of digital health applications (DiGA), students practice the basics of reading and summarizing scientific publications, independent source research, and reflection and discussion of the results.

Performance is assessed on a pass/fail basis, and various forms of feedback are used to support individual learning development. These range from sample solutions and video-based “debriefings” (instructional videos with explanations of the task) to individual written feedback in free text form or as semi-structured feedback forms. In order to evaluate learning progress in a manner, students receive between 9 and 15 items to answer at the start and after submitting the respective ePF tasks, depending on the complexity of the task. These include competence-based self-assessments that refer to the respective learning objectives. Figure 2 (Fig. 2) shows an example of the didactic approach of such a portfolio task.

### Seminars

In addition to the ePortfolio tasks, seminars have been and continue to be developed that deepen methodological aspects (e.g. scientific writing, presentation techniques, data literacy, etc.) but also aim to fill gaps in medical education, e.g. on exclusion and racism in the healthcare system [25] or on fields of application for artificial intelligence or robotics [26]. A total of 16 hours of seminars must be completed. *Blended learning* formats are used in some seminars to reduce attendance time in favour of independent learning processes. 

### Research paper

At the end of the science module, the central component is the “research paper”. This should be 10-15 pages long and not exceed a processing time of six weeks, whereby a block processing time is possible as well as preparation throughout the semester, as there is no additional free time slot specifically for research work in the current curriculum. In total, the workload should not exceed 200 hours. Students are free to choose their topic from any field of medicine, and there are no restrictions on methodology. It is possible to link the content to a subsequent or ongoing doctoral thesis, although the research paper will be assessed as an independent piece of academic work. The structure should be that of a published paper (introduction – methods – results – discussion), including a half-page abstract.

The supervisors use a standardised assessment form as a basis for their evaluation, which specifies six formal aspects (including spelling and correct referencing of sources) and seven content-related aspects (including thematic delimitation, presentation of results, discussion of results). The research paper is graded on a maximum of 33 points. An online database has been developed for the allocation of topics, in which the supervisors post their offers. After a brief review of the formal requirements by the teaching staff, the work is activated via ILIAS. If interested, students can contact the supervisors directly by email. Independently of this, students can develop and work on their own topics at and find a supervisor. Supervisors of research papers must have at least a doctorate.

### Scientific progress test

**Figure 3:**
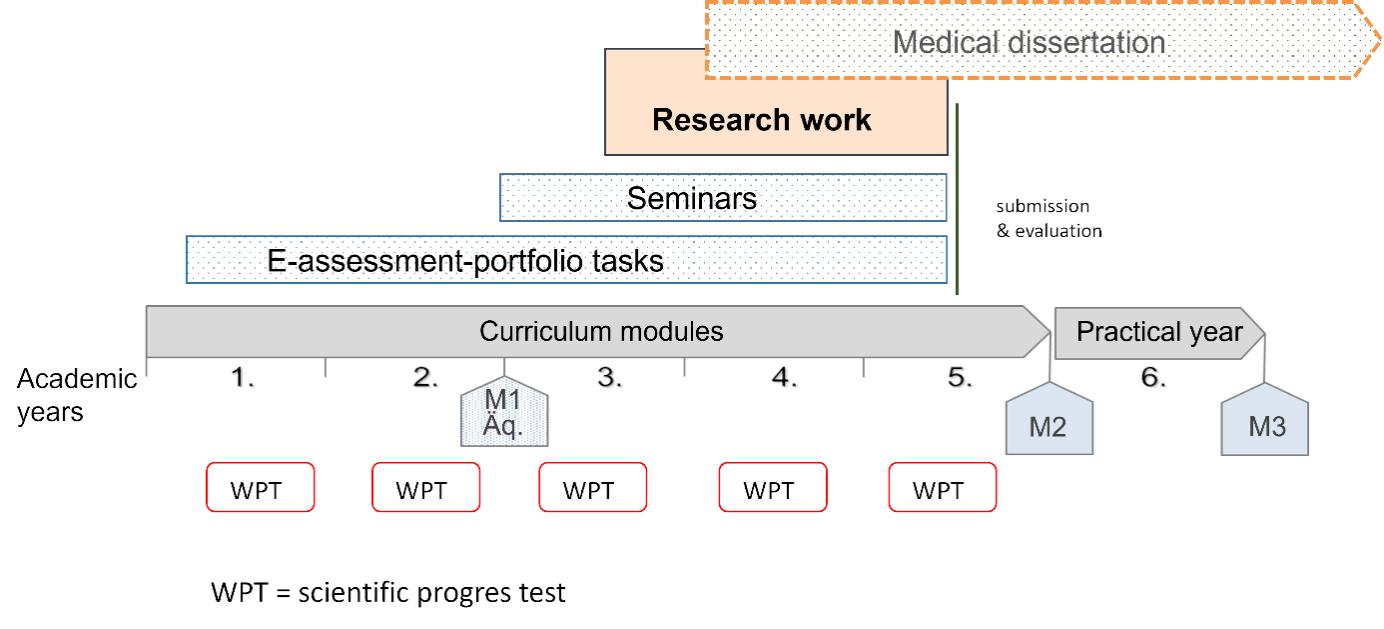
Schematic flow chart of the longitudinal science module in academic years 1-5 A workload of 60 hours is assigned to the eAssessment portfolio tasks. Seminars must be documented to a total of 16 hours. The research work should not exceed 200 hours.

An annual student progress test with currently 30 questions accompanies the entire learning process. The question pool, which contains around 200 questions, includes various question types such as long menu, matching, fill-in-the-blank and image questions. It was created primarily by students and reviewed by lecturers through an internal review process [27]. Figure 3 (Fig. 3) summarises all elements of the science module in the course of study. 

## Methods

The results on learning gains are based on data that is continuously collected as performance or pass marks as part of module administration. For the ePFA, the individual tasks recorded for two cohorts for the first two years of study were summarised as “all tasks passed” vs. “not all tasks passed”. In addition, the competence assessments collected before and after the tasks were completed, which are based on the learning objectives of the tasks, were evaluated descriptively. The items contain 5- or 7-point scales, which represent values ranging from “fully agree” to “do not agree at all”. To assess the learning gains achieved, item means and the delta (pre-post) for individual items are reported. In addition, the “total learning gain” for a task was calculated as the mean of all items in a task and the delta (pre-post) was calculated. This learning gain was tested for statistical significance using the t-test for independent samples, with p≤0.05 as the threshold value. Due to the large number of cases and the generally single-peaked value distributions for the individual items, a parametric test was chosen. Cohen’s d was calculated as a measure of the effect sizes achieved for the mean differences.

With regard to the student’s research papers available to date, the recorded (graded) results are presented. In addition, this article presents selected feedback from a student survey on their experiences with the research work. Students who submitted their research paper and received their assessment are given access to an online evaluation form that asks about key aspects of the work phase, satisfaction with the supervision and perceived learning outcomes. N=84 responses were included in the analysis. For this presentation, the information on processing time, preparation and support provided by the supervisors was analysed as closed items. In addition, the open question about the three most important learning outcomes, which was to be answered in bullet points, was evaluated using content analysis. 

## Results

### Learning progress as reflected in the ePortfolio tasks 

The ePF tasks are carried out and subjective assessments of competence gains are collected via ILIAS. The completion of individual tasks is assessed as “pass” or “fail” and allows the individual status of students in relation to the science module to be tracked. A follow-up period is granted for failed assignments. In the event of delays in study progress (due to illness, leave of absence, exchange programs), the assignments can be made up. Looking at the ongoing module performance, it can be seen that around 90% of students in the first two cohorts successfully completed the tasks within the specified time frame (based on the first six ePF tasks that had to be completed within the first two years of study).

The students' self-assessments also serve as an indicator of the content-related and methodological skills they aim to acquire through the assignments. So far, data has been collected for at least one assignment per academic year, with more in the pipeline. Self-assessments have already been collected from several cohorts for the assignment in the second academic year. The learning gains – calculated as the difference between the mean values – and the effect sizes (Cohen’s d) show that, on average, there is a substantial and statistically significant increase for all tasks, although the items within the respective tasks – i.e. the different dimensions of the skills to be assessed – can show considerable variation. Table 1 (Tab. 1) shows selected items with the minimum and maximum learning gains for each task, as well as the learning gains calculated across all items in a task.

### Feedback creation

Students receive individual feedback forms for individual assignments and group feedback forms for group assignments. Initially, these were created in free text form and supplemented with differentiated text modules, but semi-structured feedback forms are now used. These allow for a division of labour in the assessment process: student assistants check the formal aspects of the submissions in advance. The content is reviewed by lecturers from the science module. Since the question of the e feasibility of this format depends largely on the time resources available, the human and time resources invested were recorded as part of the initial task feedback. Depending on the complexity of the assignment, the average time required to provide feedback is between 15 and 30 minutes. If one person were to carry out the corrections alone, this would correspond to one third of the annual working time of a full-time research assistant (postgraduate/postdoc).

### Results of the research paper analysis

The introduction of compulsory written academic work is still uncharted territory in medical studies. For this reason, the experiences gained at the MHH to date are reported below. To this end, the first 200 or so papers submitted are classified according to subject matter and content, and the results of a student evaluation are presented. 

With regard to the choice of methods, it can be seen that the majority of the papers are written as literature reviews (56%). Around 24% stated that they had based their paper on existing data (clinical data, laboratory data, survey data). A further 20 % reported that they had collected data for their research work, which may also form the empirical basis of their doctoral project. However, more than half of this group stated that they had collected the data primarily for their research paper.

Differentiated feedback was provided with regard to the preparation for the research work and the timing of the desired support. While around one third needed support in narrowing down the research question in the run-up to the work, methodological aspects and the written elaboration were primarily perceived as difficult during the work process (see table 2 (Tab. 2)). 

**Figure 4:**
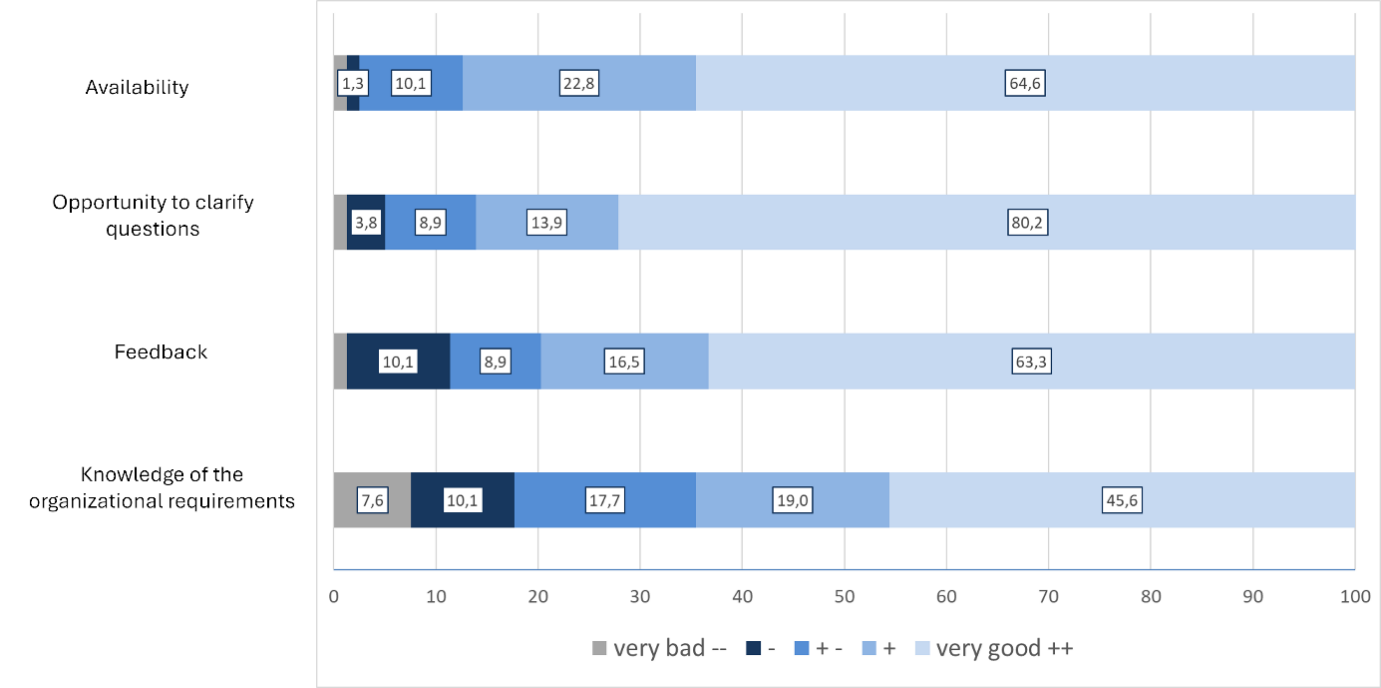
“How would you rate the following aspects of care?” (N=81): Relative and absolute frequencies. Due to rounding, the (cross) total may exceed 100%.

Since the research work is also a new format for the vast majority of teachers, satisfaction with the supervision provided was also surveyed (see figure 4 (Fig. 4)). Only a minority expressed clear dissatisfaction. This mainly concerned support with formal aspects of the work (including the registration and assessment process), while the academic guidance was largely perceived as very good or good.

Regardless of the students’ assessment, an ex-post review of the formal characteristics of the submitted papers showed that the required abstract was missing in around one third of the papers – this also indicates that the requirements for the research paper are not yet sufficiently known among the supervisors.

**Figure 5:**
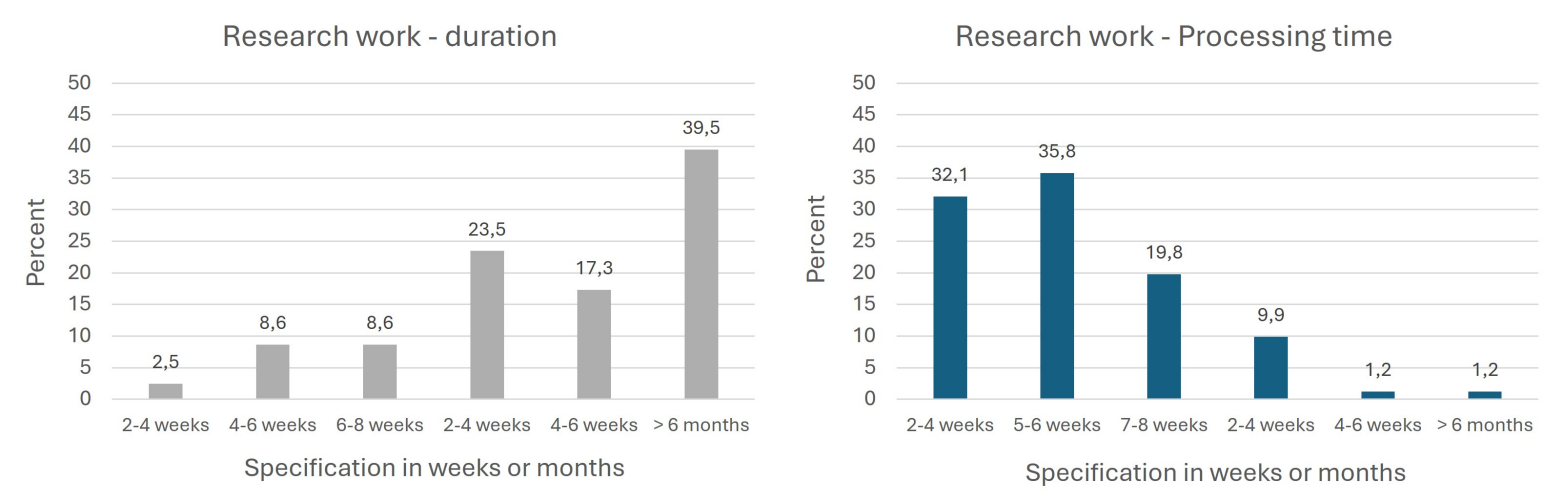
Duration of research work (time from assignment of topic to submission) & pure working time (=40 hours/week); n=81 5a=duration, 5b=processing time

With regard to the time dimension of the research work, both the pure working time and the duration were surveyed. The results showed that around two-thirds were able to meet the target of six weeks for completion (see figure 5 a (Fig. 5)) – however, this was usually spread over a longer period, mostly as semester-long phases of varying duration and intensity (see figure 5 b (Fig. 5)).

While the e-PA tasks are intended to stimulate and support learning processes as an ungraded, formative element in the science module, the research paper is graded. The standardised feedback form allows the usual grading scheme (very good – good – satisfactory – adequate – fail) to be used. In fact, the grades awarded for the first N=210 papers show little discrimination: 74% received a grade of “very good”, 22% were graded “good”, and 4% were graded “satisfactory” or “sufficient”.

**Figure 6:**
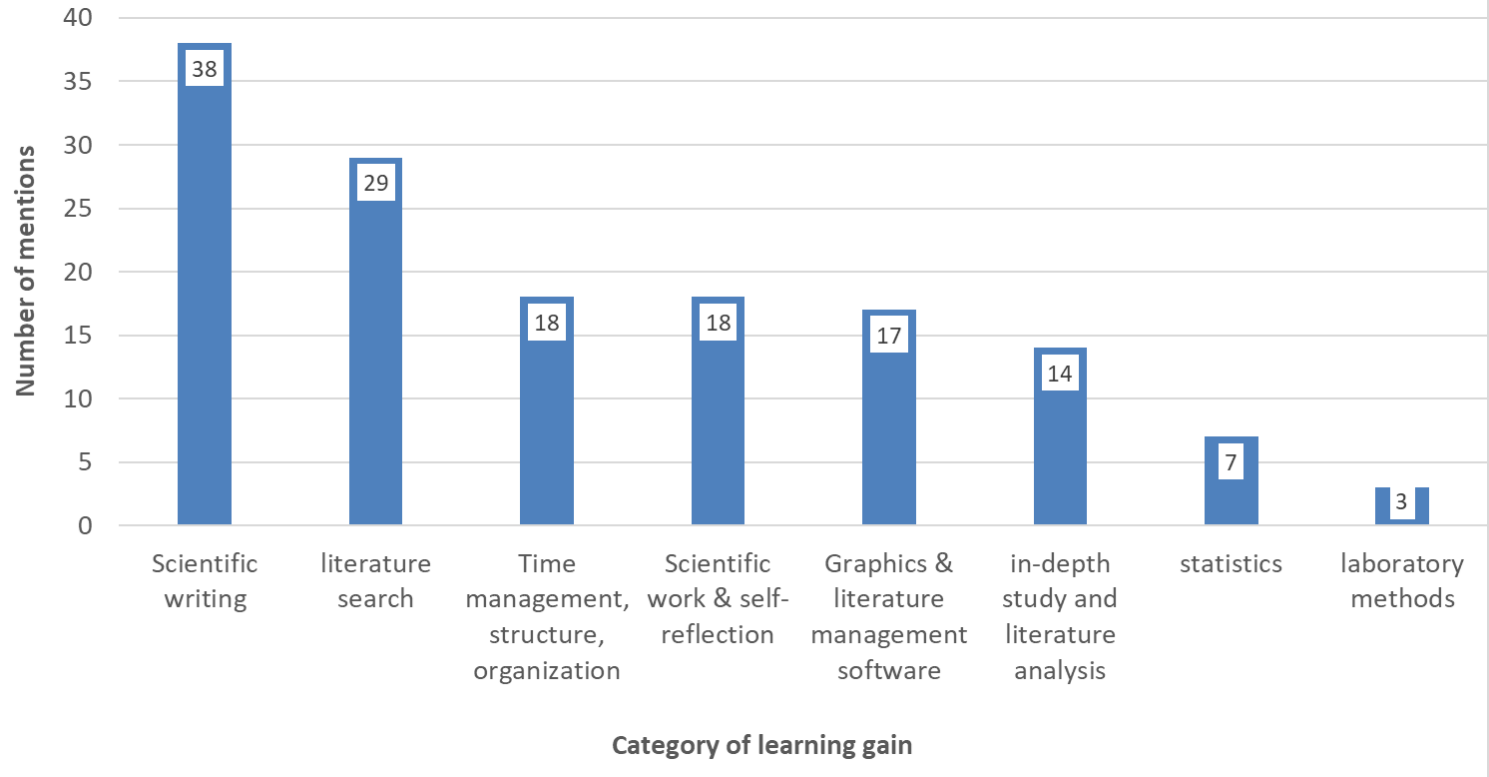
Self-assessed learning gains in the context of research work – content categories (based on free-text mentions from n=61 students with n=144 aspects)

N=61 students answered the question about the most important things they learned during their research work. The brief answers were recorded quantitatively and evaluated using content analysis. The categories were formed inductively, based on central NKLM learning objectives for scientific skills and scientific project work. Figure 6 (Fig. 6) shows the frequency distribution of the answers, with a total of n=144 separate aspects listed. It is clear that the students primarily acquire skills in scientific writing, but also in searching for and processing scientific sources.

## Discussion

With the introduction of the science module as part of the model curriculum, the MHH broke new ground in 2020. Following a pilot phase in which participation was voluntary, individual didactic elements were further developed and the module was implemented as a compulsory part of the curriculum. The implementation was based on NKLM mapping and specifically filled gaps in the existing curriculum. The concept includes eAssessment portfolio tasks with formative feedback, in-depth seminars and a final research paper. With a view to the total of 480 hours for a compulsory research project envisaged in the amendment to the licensing regulations and the longitudinal structure, the foundation for further expansion has already been laid. With regard to the learning objectives of the science module, the fundamentals anchored in the NKLM as “professional scientific activities” were predominantly used, as these had not been sufficiently taught in other existing modules at the MHH to date. Although a new licence-based study reform has not yet been agreed upon, other faculties have already significantly expanded their scientific strands and anchored them longitudinally [2], [6], [7], [8], [9]. There are considerable differences between the faculties in terms of didactic implementation, but there are also comparable elements. In principle, most medical curricula now offer preparatory courses that introduce students to scientific work. These usually take the form of seminars, practical courses or lectures. At the MHH, the basics of scientific work are taught in part as part of the ePF tasks. Students deepen and reflect on the material in the form of written assignments. This is also intended to provide practice in written formats that are otherwise rarely used in medical studies. 

The results so far show that the tasks generate significant learning gains. Although these gains vary when comparing different tasks, they are substantial in all academic years and for almost all task elements. These learning gains have also been confirmed by the results of the annual scientific progress tests available to date [27]. These results show that the selection of topics addresses and closes existing gaps in the curriculum. With the development of semi-structured written feedback, elements that are rarely found in medical education have also been tested. Formative feedback is considered by students to be an important – yet often neglected – teaching resource [28]. Surveys have shown that medical students are the most dissatisfied with feedback on their performance compared to other subjects [29]. Our experience with ePF tasks to date has shown that reviewing and providing feedback is more resource-intensive than standardised MC exams. However, individual feedback allows for more accurate feedback on students' strengths and areas for improvement. This approach still has potential for development: for example, it could be further developed towards adaptive tasks: students who demonstrate mature methodological and reflective skills at an early stage in the ePortfolio tasks could be introduced to the final research project in a targeted and earlier manner. 

The increased time required to correct student submissions nevertheless poses a challenge for teachers, especially as many medical faculties are increasing the admission capacity. In addition to being reflected as a quantitative teaching achievement, crediting formative feedback as a qualifying element for academic advancement (Habilitation or adjunct Professor) could also increase its attractiveness. However, it remains to be seen whether and to what extent formative feedback itself can contribute to increased learning or motivation to learn. Future evaluations should attempt to address this question.

The supervision of research projects aimed at developing basic scientific competencies remains unfamiliar to many faculty members. Unlike medical dissertations, which often result in a publication, the research paper is a study achievement that is not (primarily) aimed at producing new scientific findings. However, the first 200 or so completed projects have shown that introducing a six-week scientific project can have a number of positive effects. The learning gains reported by the majority of students as a result of the research work cover a range of corresponding learning objectives of the NKLM that were not previously anchored in the curriculum in any depth. These include, first and foremost, the adequate written, graphical and tabular presentation of scientific findings (VIII.1-04.2.; VIII.1-04.1.12; VIII.1-04.2.7). The development of basic skills within the framework of individual projects (VIII.1-04.2.2) also leads to a range of additional skills that offer added value both for further research paths and for everyday medical work: in particular, literature search and the critical reception of study results (VIII.1-03.1.4; VIII.1-04.2.1; VIII.1-02.1.4). In the context of these processes, the more skilled use of software essential for text and graphic production and literature management should also be noted.

Further information provided by the students indicates – albeit to a lesser extent – an increase in competence in data analysis methods and statistical methods. Another important aspect is the opportunity for students to familiarise themselves with the scientific community. This includes discussions with researchers and doctors, lectures, dealing with feedback, but also self-reflection on one's own strengths and weaknesses (VIII.6-03.1.5; VIII.6-03.1.1; VIII.1-02.1.1).

Nevertheless, conceptual deficits have also become apparent in the introductory process to date. On the one hand, the level of information available to students and supervisors about the formal and didactic requirements of the research paper is sometimes incomplete. This becomes apparent not only through the failure to adhere to formal requirements but also through the frequent inquiries by students regarding information already available to instructors in ILIAS. A look at the evaluation of the quality of supervision, but also at the variation in the amount of time spent, shows that a uniform standard in terms of the scope and supervision of the work has not yet been achieved. 

Regardless of the goal of academic advancement, the quality of supervision is a central component of the concept of academic training [9]. Since medical and academic staff from different disciplines are usually involved, establishing binding standards remains a central challenge. At the MHH, the supervision of qualification theses is therefore addressed in courses on good academic practice for lecturers. Attendance at this course is mandatory for obtaining the* venia legendi* (Habilitation). However, guiding students, assisting them in developing questions and writing their papers can also provide impetus for one's own qualification during the medical postgraduate training. Further research is needed on this aspect of “secondary” scientific competence growth in the context of continuing education or career development. 

From the students' perspective, the analyses presented here show that the tasks to be completed and the final research project offer learning gains – as reflected in their self-assessments. Future studies must show whether these effects can be sustained over the entire duration of the programme and beyond. The question of which teaching formats and concepts are particularly suitable for strengthening scientific skills in medical studies also remains open for the time being. In particular, the possible uses of generative artificial intelligence raise new questions in this context, which would go beyond the scope of this paper to discuss in detail. From the perspective of those responsible for teaching the science module, the approach of “critical use” has proven successful so far. An MHH guideline on “dealing with text-generating artificial intelligence (AI) in the creation of scientific documents” obliges students to disclose the use and extent of AI in the context of their work. So far, we are not aware of any cases in which this has been used, nor have many cases of misuse come to light.

For the time being, other criteria seem to be more important for the success or failure of a limited, written scientific paper: in critical feedback on the module, students mostly criticise the additional time required for research work. For the permanent integration of the approaches tested so far into the curriculum, it is very important that they are implemented in new licensing regulations and that protected time for academic work is expanded during studies: as a signal to medical faculties and to secure the necessary human resources for strategic and content-related further development. 

## Conclusion and outlook

The further development of the NKLM and the mapping of the existing curriculum at the faculty will enable the development of tailored academic curricula while avoiding redundancies. The introduction of new, science-oriented teaching and learning concepts provides important impetus for future-oriented medical education. They offer students greater freedom of choice, for example through the selection of a research topic during their studies, which strengthens their own interests and thus their motivation. At the MHH, the science module was developed with the aim of opening up new perspectives on the highly school-based structure of medical studies through independent learning processes. This is achieved on the one hand through ePortfolio tasks, which can be used to address a wide range of fundamental as well as current topics. As in other faculties, a research paper at the MHH concludes the science module. Closer supervision in the context of this project work allows students to experience scientific work in a real research context – similar to their experiences in everyday clinical practice. Conversely, clinics and institutes have the opportunity to involve research-oriented young talent at an early stage, train them and thus bring them into research. Conversely, clinics and institutes can capitalize on the opportunity to engage research-oriented students at an early stage, provide structured training, and thereby facilitate their systematic integration into research activities

## Funding

The development of the science module was funded by third-party funds from the programmes “Innovative Teaching and Learning Concepts: Innovation Plus” and “Quality Plus – Programme for Good Teaching in Lower Saxony” of the Lower Saxony Ministry of Science and Culture (MWK).

## Authors’ ORCIDs

- Volker Paulmann: [0000-0002-9143-2794]
- Marie Mikuteit: [0000-0001-8546-0548]
- Ingo Just: [0000-0002-8176-8534]
- Sandra Steffens: [0000-0002-7478-3920]

## Competing interests

The authors declare that they have no competing interests. 

## Figures and Tables

**Table 1:**
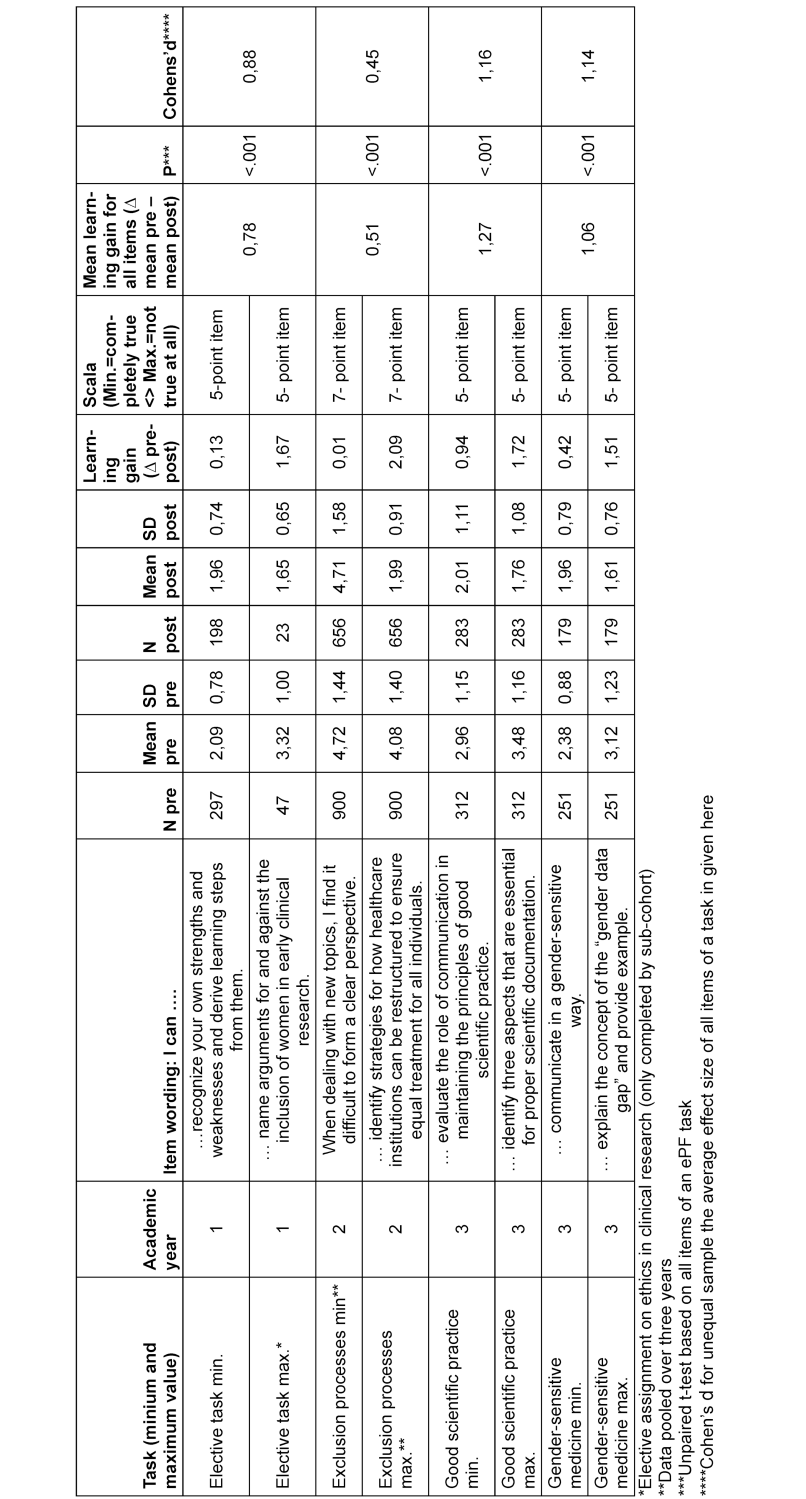
Assessment of learning gains for ePortfolio tasks from the first three years of study For each ePortfolio (ePF) task, between 9 and 15 items were provided for completion before and after task processing. The scale was based on five or seven levels with the poles “Min.=fully applies” <> “Max.=does not apply at all.” Shown are the minimum and maximum learning gains for items from each ePF task, as well as the overall learning gain for all items of a task based on the delta (pre–post). Statistical significance was determined using the t-test for unpaired samples on the basis of the pre–post items of the respective task. As an effect size estimator, Cohen’s d was calculated (here as the average of the learning gain effect sizes of the items of a task). Cohen suggests the following thresholds: d=0.20 (small effect), d=0.50 (medium effect), and d=0.80 (large effect).

**Table 2:**
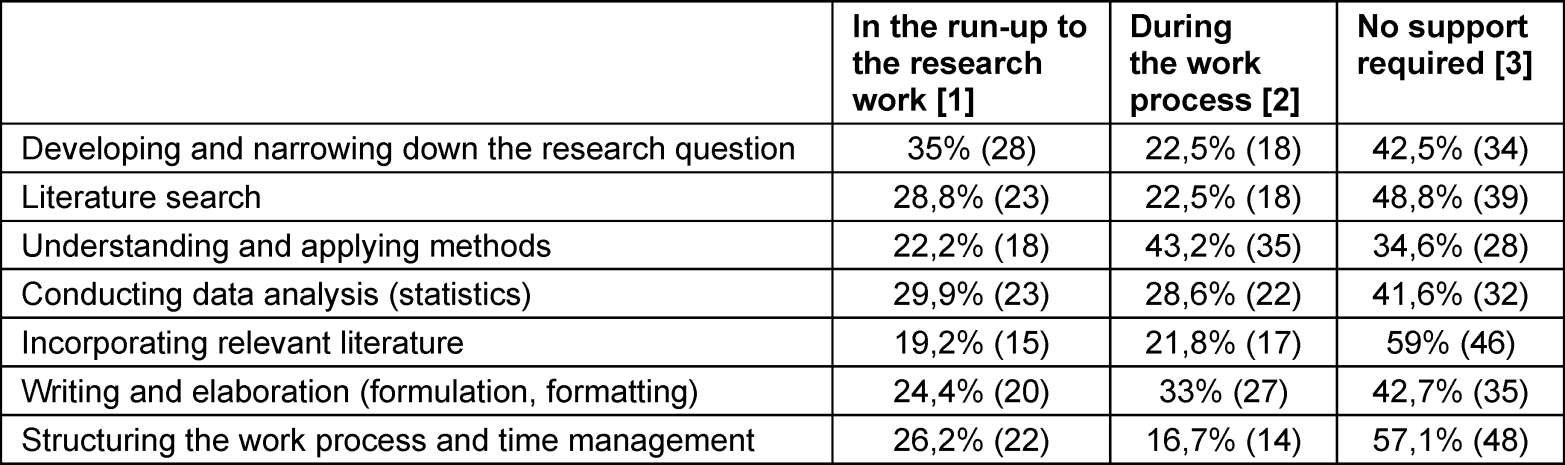
“To what extent would you have liked more support” (N=81): Relative and absolute frequencies Due to rounding, the (cross) total may exceed 100%.
